# Spontaneous eye blink rate and dopamine synthesis capacity: preliminary evidence for an absence of positive correlation

**DOI:** 10.1111/ejn.13895

**Published:** 2018-03-26

**Authors:** Guillaume Sescousse, Romain Ligneul, Ruth J. van Holst, Lieneke K. Janssen, Femke de Boer, Marcel Janssen, Anne S. Berry, William J. Jagust, Roshan Cools

**Affiliations:** ^1^ Donders Institute for Brain, Cognition and Behaviour Donders Centre for Cognitive Neuroimaging Radboud University PO Box 9101 Nijmegen 6500 HB The Netherlands; ^2^ Department of Psychiatry Academic Medical Center University of Amsterdam Amsterdam The Netherlands; ^3^ Department of Neurology Max Planck Institute for Human Cognitive and Brain Sciences Leipzig Germany; ^4^ Social, Health, and Organizational Psychology Utrecht University Utrecht The Netherlands; ^5^ Department of Nuclear Medicine Radboudumc Nijmegen The Netherlands; ^6^ Helen Wills Neuroscience Institute University of California, Berkeley Berkeley CA USA; ^7^ Department of Psychiatry Radboudumc Nijmegen The Netherlands

**Keywords:** [^18^F]DOPA, dopamine, eye blink rate, PET, proxy measure

## Abstract

Dopamine is central to a number of cognitive functions and brain disorders. Given the cost of neurochemical imaging in humans, behavioural proxy measures of dopamine have gained in popularity in the past decade, such as spontaneous eye blink rate (sEBR). Increased sEBR is commonly associated with increased dopamine function based on pharmacological evidence and patient studies. Yet, this hypothesis has not been validated using *in vivo* measures of dopamine function in humans. To fill this gap, we measured sEBR and striatal dopamine synthesis capacity using [^18^F]DOPA PET in 20 participants (nine healthy individuals and 11 pathological gamblers). Our results, based on frequentist and Bayesian statistics, as well as region‐of‐interest and voxel‐wise analyses, argue against a positive relationship between sEBR and striatal dopamine synthesis capacity. They show that, if anything, the evidence is in favour of a negative relationship. These results, which complement findings from a recent study that failed to observe a relationship between sEBR and dopamine D2 receptor availability, suggest that caution and nuance are warranted when interpreting sEBR in terms of a proxy measure of striatal dopamine.

## Introduction

Dopamine is a key ascending neuromodulator subserving a range of cognitive functions, including motivation, learning and attention. In turn, striatal dopamine dysregulation is a common feature across many psychiatric disorders in which those functions are impaired, including addiction, schizophrenia, attention deficit hyperactivity disorder and depression (Russo & Nestler, 2013; Nutt *et al*., 2015; Grace, 2016). Unfortunately, direct assessment of dopamine function in humans is only possible with expensive and invasive techniques such as positron emission tomography (PET), which require complex infrastructure and in‐depth methodological expertise. As a consequence, non‐invasive proxy measures have gained in popularity. In particular, spontaneous eye blink rate (sEBR) has been used in many studies to investigate dopamine's role in various cognitive tasks and patient populations (for a comprehensive review see Jongkees & Colzato, 2016).

Evidence supporting a link between dopamine function and sEBR comes primarily from pharmacological studies in both humans and animals. These studies have shown that dopamine‐enhancing drugs generally increase sEBR (Karson, 1983; Strakowski *et al*., 1996), whereas dopamine‐decreasing drugs tend to decrease sEBR (Lawrence & Redmond, 1991; Mavridis *et al*., 1991) – although a number of other studies have reported null or opposite results (Ebert *et al*., 1996; Kleven & Koek, 1996; van der Post *et al*., 2004; Mohr *et al*., 2005; Kotani *et al*., 2016). Similarly, brain disorders characterized by a hypo‐dopaminergic state, such as Parkinson's disease, have been mostly associated with decreased sEBR (Deuschl & Goddemeier, 1998; Chen *et al*., 2003), whereas disorders characterized by a hyperdopaminergic state, such as schizophrenia, have been associated with increased sEBR (Chen *et al*., 1996; Chan *et al*., 2010). These observations have led to the pervasive hypothesis that sEBR is positively correlated with dopamine function (Jongkees & Colzato, 2016).

However, establishing sEBR as a reliable marker of human dopamine function requires direct evidence for a relationship between sEBR and *in vivo* measures of dopamine function in humans. Dopamine function can be measured in several ways, for example, in terms of synthesis capacity, transporter density and/or (changes in) receptor availability. Only two PET studies to date have tackled this question, and both of these focused on dopamine receptor availability. The first study by Groman *et al*. (2014) measured dopamine D2 and D1 receptor availabilities in vervet monkeys, using [^18^F]fallypride and [^11^C]NNC‐112 PET, respectively. The results showed that striatal dopamine D2 (but not D1) receptor availability was positively correlated with sEBR, as well as with D2 receptor agonist‐induced changes in sEBR. However, a recent study by Dang *et al*. (2017) was unable to replicate these results in humans. Using [^18^F]fallypride PET and the dopamine D2 receptor agonist bromocriptine, they found that dopamine D2 receptor availability was neither related to baseline sEBR or bromocriptine‐induced changes in sEBR. In this study, we focused on another aspect of dopamine function, namely striatal dopamine synthesis capacity, as measured by [^18^F]DOPA PET. Based on the above‐described literature and the commonly assumed positive relationship between sEBR and dopamine function (Jongkees & Colzato, 2016), we hypothesized that dopamine synthesis capacity would be positively related to sEBR. This hypothesis is further in line with a previous report showing that sEBR is positively correlated with post‐mortem measures of dopamine concentration in the caudate nucleus of monkeys treated with the neurotoxic drug MPTP (Taylor *et al*., 1999).

## Materials and methods

### Participants

Thirty male participants were recruited, among which were 15 pathological gamblers (PGs) and 15 matched healthy controls (HCs). The PET data from these participants have been reported in a previous study comparing baseline dopamine synthesis capacity between groups (van Holst *et al*., 2017). PGs were recruited through advertisement and addiction treatment centres and reported not to be medicated or in treatment for their gambling at the time of the PET study. HCs were recruited through advertisement. All gamblers qualified as PGs as they met ≥ 5 DSM‐IV‐TR criteria for pathological gambling. Current drug use disorder was a reason for exclusion for all participants. Additionally, participants were excluded if they were currently following psychiatric treatment, drank more than four alcoholic beverages daily, used marijuana more than once per month, were using (psychotropic) medication, had a lifetime history (as assessed with the MINI interview) of schizophrenia, bipolar disorder, attention deficit hyperactivity disorder, autism, bulimia, anorexia, anxiety disorder, obsessive–compulsive disorder or had a past 6‐month history of major depressive episode.

Nine participants (three PGs; six HCs) were not included due to technical problems with the electro‐oculography (EOG) recordings (data loss > 50% of the total recording time). Data loss was related to EOG amplifier saturation (output voltage of the amplifier exceeding its operating range sensitivity). Three participants were kept in the analyses despite minor data loss (exploitable data: 3.5, 4.4 and 4.5 min). One gambler was further excluded because he met the DSM‐IV‐TR criteria for past year marijuana dependence, which is known to influence sEBR measures (Kowal *et al*., 2011). As a result, the final sample comprised 20 participants (mean age: 38.2, age range: 22–54, gamblers: *n* = 11, controls: *n* = 9).

All participants provided written informed consent to take part in the study, which was approved by the regional research ethics committee (Commissie Mensgebonden Onderzoek, region Arnhem‐Nijmegen, registration number NL41522.091.12).

### Study procedures

The study took place at the Radboud University Medical Center. Upon arrival of the participants, spontaneous eye blink rate (sEBR) was measured using EOG recordings (see detailed description below). Approximately 1 h before entering the PET scanner, participants received 150 mg of carbidopa and 400 mg of entacapone to reduce peripheral metabolism of [^18^F]DOPA and increase tracer availability in the brain while having no psychotropic side effects. The participants further performed computerized tasks not reported here.

### Spontaneous eye blink rate: recording and analyses

We followed standard procedures for the acquisition, preprocessing and analysis of the EOG data (Colzato *et al*., 2009; Slagter *et al*., 2010). The data were acquired during the day (3:30–4:30 pm) over a 6‐min period, using two vertical and two horizontal Ag‐AgCl electrodes placed around the left eye. The sampling rate was 100 Hz. The vertical EOG signal (vEOG), used for eye blink assessment, was obtained from a bipolar montage using the electrodes placed above and below the eye. The horizontal EOG (hEOG) signal, used to exclude artefacts produced by saccades, was obtained from a bipolar montage using the electrodes placed lateral to the external canthi.

Participants were comfortably seated and instructed to fixate the wall in front of them while they believed the system was being calibrated and the experimenter was outside of the room. They were not instructed in any way about blinking and were asked not to move their head or activate their facial muscles to minimize EOG artefacts.

The vEOG signals were rectified and band‐pass filtered between 0.5 and 20 Hz. Eye blinks were detected using an automated procedure based on a voltage change threshold of 100 μV in a time interval of 400 ms. The vEOG signal was then visually inspected by two of the authors (R.L. and G.S.) to assess detection accuracy (i.e. presence/absence of blinks) and remove potential artefacts resulting from muscle activity and saccades as detected in the hEOG signal. As the inter‐rater reliability was very high (Cronbach's α = 0.989), the resulting sEBR measures from the two raters were averaged for subsequent analyses.

### PET and MRI data acquisition

All PET scans were acquired at the department of Nuclear Medicine of the Radboud University Medical Center using a Siemens mCT PET/CT camera (with 40 slice CT, voxel size 4 × 4 mm in‐plane, 5 mm slice thickness). Participants were positioned as comfortably as possible, in a supine position, with the head slightly fixated in a headrest to avoid movement. First, a low‐dose CT was made for attenuation correction of the PET images, followed by an 89‐min dynamic PET scan. The scan started at the same time as the bolus injection of the [^18^F]DOPA into an antecubital vein. Images were reconstructed with an ordered subset expectation maximization algorithm with weighted attenuation and time‐of‐flight recovery, scatter corrected and smoothed with a 4‐mm FWHM kernel.

A high‐resolution anatomical scan (T1‐weighted MP‐RAGE, TR = 2300 ms, TE = 3.03 ms, 8° flip‐angle, 192 sagittal slices, slice‐matrix size = 256 × 256, voxel size 1 × 1 × 1 mm^3^) was obtained using a 3 Tesla MR Siemens scanner at the Donders Centre for Cognitive Neuroimaging and used for co‐registration with the PET data.

### PET data preprocessing and analyses

**Figure 1:**
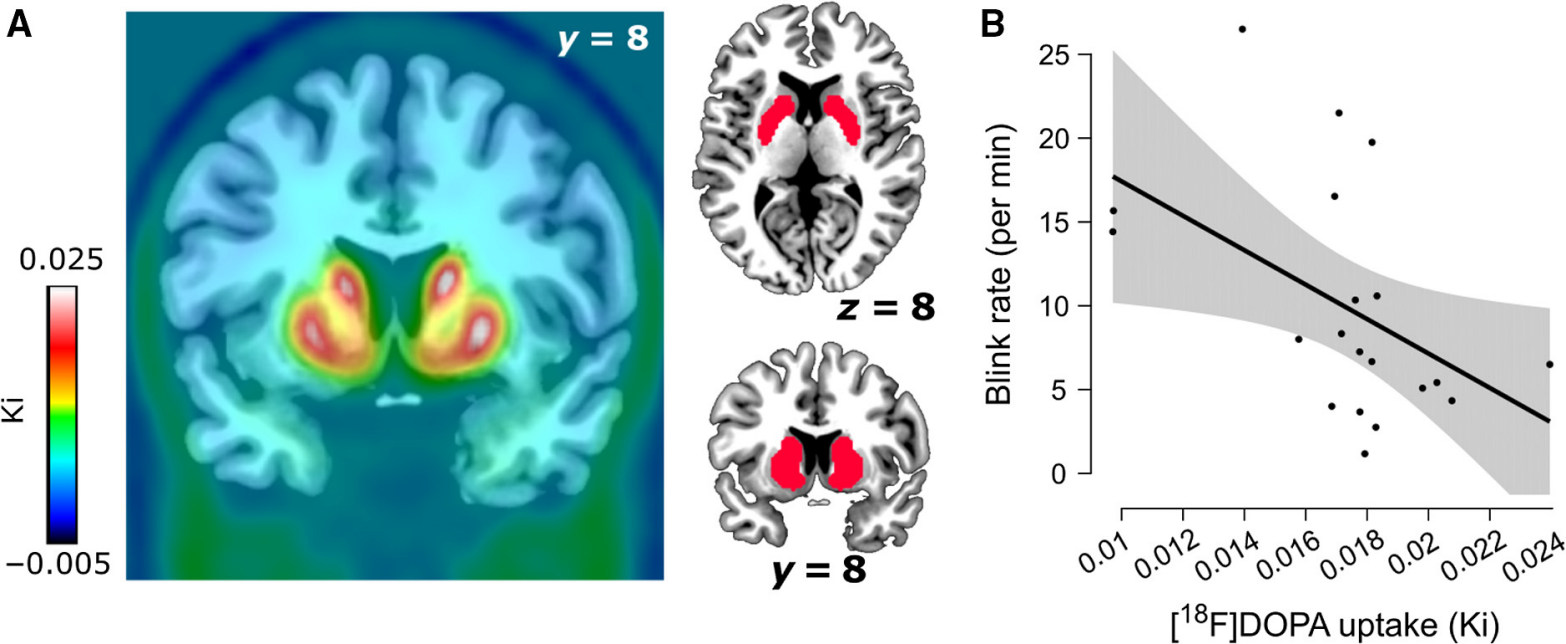
Main results: mapping of dopamine synthesis capacity and relationship with spontaneous eye blink rate in the striatum. (A) On the left, whole‐brain map of mean Ki values across the 20 participants included in the analyses. On the right, the striatal functional mask used for the region‐of‐interest analyses is depicted in red. (B) Negative correlation between spontaneous eye blink rate and Ki values in the striatal mask (⍴ = −0.504, *P* = 0.024). The shaded area represents the 95% confidence interval. Note that the *P*‐value of the negative correlation drops below significance (⍴ = −0.417, *P* = 0.096) when removing the three most extreme Ki values (highest Ki value and two lowest Ki values, respectively 2.1 and 2.4 standard deviations away from the mean). [Colour figure can be viewed at http://www.wileyonlinelibrary.com/].

We realigned the [^18^F]DOPA images to the middle (11th) frame to correct for movement during scanning using SPM8 (http://www.fil.ion.ucl.ac.uk/spm/). The mean [^18^F]DOPA image and the realigned frames were coregistered to the structural MRI scan using SPM8. Higher [^18^F]DOPA uptake, indexed by higher Ki values, is established to reflect higher dopamine synthesis capacity (Snow *et al*., 1993). To create Ki images representing the amount of tracer accumulated in the brain relative to a cerebellar region of reference, we used an in‐house graphical analysis program implementing Patlak plotting (Patlak & Blasberg, 1985). Ki images were generated from PET frames corresponding to 24–89 min. These images are comparable to Ki images obtained using a blood input function but are scaled to the volume of tracer distribution in the reference region. Finally, structural MRI scans were normalized to a standard MNI template (IBASPM152), and the transformation matrix was applied to coregistered Ki images. As expected from previous studies (Rakshi *et al*., 1999), the voxels with the highest Ki values were located in the striatum and the brainstem (Fig. 1A).

For the main analyses, Ki values were extracted from an independently and functionally defined striatal region of interest, based on a whole‐brain [^18^F]DOPA PET template normalized to MNI space (García‐Gómez *et al*., 2012, downloaded from http://www.nitrc.org/projects/spmtemplates). Specifically, we retained the template voxels in which Ki values were at least three standard deviations above the template mean, thus approximating the anatomical boundaries of the striatum (in which [^18^F]DOPA binding is maximal, see Fig. 1A).

**Figure 2:**
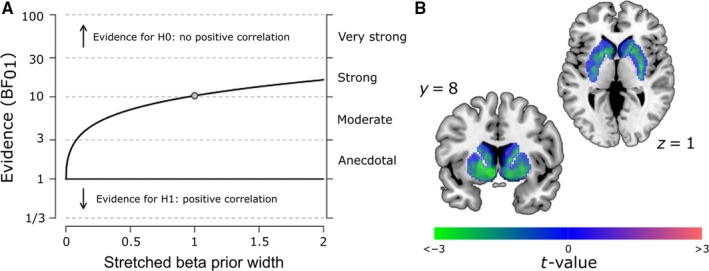
Control and exploratory analyses. (A) Robustness check for the Bayesian correlation analysis. This graph shows that the Bayes factor quantifying the relative evidence for the absence vs. presence of a positive correlation exceeds the critical threshold of 3 for a large range of beta prior widths, even extending to strong prior beliefs in the existence of a positive correlation (values < 1 correspond to a prior biased in favour of a positive correlation, values > 1 correspond to a prior biased in favour of an absence of a positive correlation and value = 1 corresponds to an uninformative (flat) prior as used in our main analysis). (B) Unthresholded *t*‐map resulting from a voxel‐wise analysis investigating the relationship between sEBR and Ki values in an anatomical mask of the striatum. Note that almost all *t*‐values (including those that do not reach statistical significance) are negative, illustrating that the relationship between sEBR and dopamine synthesis capacity is negative across a large portion of the striatum. This negative relationship shows a significant peak in the left nucleus accumbens (*x*,* y*,* z* = −8, 10, −6), surviving a voxel‐wise FWE‐corrected threshold of *P* < 0.05 within the striatal mask. [Colour figure can be viewed at http://www.wileyonlinelibrary.com/].

For the exploratory voxel‐wise analysis (Fig. 2B), we performed a linear regression using SPM8. The analysis was restricted to an anatomical mask covering the entire striatum (Choi *et al*., 2012), within which the results were corrected for multiple comparisons using a voxel‐wise family‐wise error (FWE)‐corrected threshold of *P* < 0.05.

### Statistics

As extracted Ki values were non‐normally distributed (Kolmogorov–Smirnov test: *P* < 0.003), we used rank correlation coefficients (Spearman ρ). We also performed partial regression analyses, in which beta coefficients were estimated using bootstrapping in spss. Bayesian analyses were conducted using jasp (Wagenmakers *et al*., 2018).

## Results

In contrast to our prediction, frequentist statistics indicated a negative relationship between sEBR and Ki values (Spearman ⍴ = −0.504, *P* = 0.024, Fig. 1B). As both age and gambling status are known to be related to Ki values (Berry *et al*., 2016; van Holst *et al*., 2017), we next regressed sEBR onto Ki values while adding these two variables as covariates, revealing a marginally significant negative relationship between sEBR and dopamine synthesis capacity (β = −1.82, *P* = 0.09).

Given the unexpectedness of this finding, we sought to quantify the evidence for the null hypothesis that sEBR and Ki values are not positively correlated (H0), as opposed to positively correlated (H1). We used a Bayesian analysis with an uninformative (flat) prior distribution over the (0, 1) interval. The relative evidence in favour of H0 over H1 was strong (Bayes factor: BF_01_ = 10.34), indicating that our data are 10 times more likely under the null hypothesis of no positive relationship than under the alternative hypothesis of a positive relationship. Importantly, a Bayes factor robustness check showed that this conclusion holds even when using strong prior beliefs in the existence of a positive correlation (Fig. 2A). In fact, there was also moderate evidence (BF_10_ = 4.90) in favour of a negative relationship between sEBR and Ki values (H1 vs. H0 defined as an absence of negative relationship).

Finally, an exploratory voxel‐wise analysis showed that the negative relationship between sEBR and Ki values was strongest in the left nucleus accumbens (Fig. 2B).

### Control analyses

To assess the robustness of our results, we performed a number of control analyses. First, we re‐ran our analyses after excluding the data from the three participants with incomplete sEBR datasets. The results remained unchanged, showing a negative correlation between sEBR and striatal dopamine syntheses (Spearman ⍴ = −0.5, *P* = 0.016), while the Bayesian correlation analysis indicated moderate to strong evidence against hypothesis H1 of a positive correlation (BF_01_ = 9.95). When splitting the data as a function of gambling status, we observed a negative association between sEBR and Ki values in both groups [healthy controls (*n* = 9): ⍴ = −0.63, *P* = 0.067; pathological gamblers (*n* = 11): ⍴ = −0.46, *P* = 0.15]. Although *P*‐values did not reach significance, Bayesian analyses similarly revealed moderate evidence against a positive correlation (healthy controls: BF_01_ = 5.66; pathological gamblers: BF_01_ = 6.29), although, of course, these values should be interpreted with caution given the small sample sizes of these subgroups.

Finally, we verified that our results were not influenced by the method used to define eye blinks. Indeed, in our main analysis, we used a relatively low detection threshold of 100 μV, which has the advantage of capturing most blinks (high sensitivity), but has the disadvantage of increasing the likelihood of false positives due to muscular artefacts (decreased specificity), which then requires manual (i.e. subjective) editing. To circumvent that issue, we re‐ran our analyses using more conservative thresholds (200 and 300 μV), which provide increased specificity and thus eliminate the need for manual editing (at the cost of decreased sensitivity). The resulting sEBR measures were again negatively correlated with Ki values, although not significantly (200 μV: Spearman ⍴ = −0.41, *P* =0.077; 300 μV: Spearman ⍴ = −0.38, *P* = 0.099), while corresponding Bayes factors again provided strong evidence in favour of the null hypothesis H0 (200 μV: BF_01_ = 10.23; 300 μV: BF_01_ = 10.29).

## Discussion

Spontaneous EBR has become increasingly popular as a putative behavioural marker of endogenous dopamine. Interestingly, most of the past studies that have used sEBR in this capacity have loosely referred to ‘dopamine function’ or ‘dopamine activity’, perhaps reflecting the current dearth of knowledge regarding which specific aspect(s) of the dopamine system correlate(s) with sEBR. Here we tested the hypothesis of a positive relationship between sEBR and striatal dopamine synthesis capacity, based on the proposal that sEBR is positively related to striatal dopamine function (Jongkees & Colzato, 2016), and previous results showing a positive correlation between sEBR and striatal dopamine levels measured in post‐mortem monkey brains (Taylor *et al*., 1999). Both frequentist and Bayesian statistics, as well as ROI and voxel‐wise analyses, argue against the existence of such a positive relationship in our sample and show that, if anything, the evidence is in favour of a negative relationship. While we prefer to refrain from making speculative interpretations regarding the existence of a negative relationship, given the moderate level of evidence, we believe these data provide a strong cautionary message against the use of sEBR as a positive predictor of pre‐synaptic striatal dopamine.

Importantly, our data speak specifically to dopamine synthesis capacity and do not preclude correlations of sEBR with other aspects of the dopamine system, including (changes in) dopamine D2 receptor availability. It has been suggested that sEBR might primarily reflect striatal D2 receptor activity, based on the observation of a positive correlation with D2 receptor availability in monkeys (Groman *et al*., 2014), and the observation that sEBR better predicts D2‐mediated punishment learning than D1‐mediated reward learning (Cavanagh *et al*., 2014; Slagter *et al*., 2015). However, this relationship between sEBR and dopamine D2 functioning has been recently questioned by a PET study which failed to replicate it in humans (Dang *et al*., 2017). Also, such a relationship remains difficult to reconcile with recent findings showing a positive association between dopamine D2 receptor availability and dopamine synthesis capacity (Berry *et al*., 2018; but see Ito *et al*., 2011, showing a negative association, although in a much smaller sample size of *n* = 14 instead of *n* = 40), as this would predict a positive relationship between sEBR and dopamine synthesis capacity, in contrast to our results. These inconsistencies certainly call for further research to better elucidate the dopaminergic mechanisms underlying sEBR.

This study is not devoid of limitations. First, our sample size (*n* = 20) is relatively small, although one should note that it is larger than the samples used in many of the psychopharmacological studies that have investigated dopaminergic drug effects on sEBR (Jongkees & Colzato, 2016). In fact, a power analysis performed with GPower3.1 (Faul *et al*., 2009) suggests that a sample size of 12 individuals should have been sufficient to replicate with 95% power the positive relationship reported by Taylor *et al*. (1999) between striatal dopamine levels and sEBR in monkeys (original effect size: *R*² = 0.62). In addition, as argued by Dang *et al*. (2017), the use of sEBR as a reliable predictor of dopamine function implicitly requires that the positive relationship between these two variables should be strong and thus observable even in small samples. For these reasons, we believe that the preliminary evidence reported here is valuable, even though a replication in a larger sample size is warranted.

Another aspect that may be perceived as a limitation is the use of a mixed population of healthy participants and pathological gamblers. While we acknowledge that pathological gamblers are not typical individuals and are characterized, among other things, by elevated striatal dopamine synthesis (van Holst *et al*., 2017), we believe that this is not necessarily an issue in the context of the current study. Indeed, our goal was to examine whether individual differences in sEBR and dopamine synthesis were positively related, regardless of the origin of these individual differences. If sEBR is to be used as proxy measure of dopamine levels, it should be insensitive to the underlying causes of individual variations, so that it can be effectively used in both clinical and non‐clinical populations. In fact, a large portion of the literature that has led to the hypothesis of a link between sEBR and dopamine function is based on the study of clinical populations characterized by dopamine dysfunctions. Finally, one should note that restricting our analyses to healthy individuals did not affect the results, still showing moderate evidence against a positive correlation.

To conclude, our study does not support the hypothesis of a positive relationship between sEBR and striatal dopamine synthesis, and if anything, provides evidence against it. Even though it is based on a modest sample size and needs to be replicated in a larger sample – which we are currently attempting to do, it warrants caution for future studies that may be tempted to use sEBR as a proxy measure of striatal dopamine synthesis capacity.

## Conflict of interest

WJJ serves as a consultant to Genentech, Novartis, and Bioclinica. All other authors report no biomedical financial interests or potential conflicts of interest.

## Data accessibility

The study data and analysis scripts are available online at https://doi.org/10.6084/m9.figshare.5878777.

## Author contributions

GS, RJvH and RC designed the study. RJvH and LKJ collected the data. GS, RL, RJvH, FdB, MJ, ASB and WJJ performed data analysis. GS and RL wrote the first draft of the manuscript. RJvH and RC edited the manuscript. We wish to thank Heleen Slagter for kindly sharing her analysis scripts for sEBR data.

**Abbreviations**
EOG: Electro‐oculography
MRI: Magnetic Resonance Imaging
PET: Positron Emission Tomography
sEBR: spontaneaous Eye Blink Rate
