# Supplementary material for: Spontaneous eye blink rate and dopamine synthesis capacity: preliminary evidence for an absence of positive correlation
Source: Eur J Neurosci. 2018 Mar 26;47(9):1081–6. doi: 10.1111/ejn.13895 (PMC5969266; doi:10.1111/ejn.13895)
Supplement: Supplementary file 1 [file EJN-47-1081-s001.pdf]

EJN European Journal of Neuroscience FENS Federation of European Neuroscience Societies

Spontaneous eye blink rate and dopamine synthesis capacity: Preliminary evidence for an absence of positive correlation

Guillaume Sescousse, Romain Ligneul, Ruth J. van Holst, Lieneke K. Janssen, Femke de Boer, Marcel Janssen, Anne S. Berry, William J. Jagust & Roshan Cools

Review timeline:

Submission date:	25 November 2017
Editorial Decision:	23 January 2018
Revision received:	17 February 2018
Accepted:	27 February 2018

Editor: Heleen Slagter

1st Editorial Decision 23 January 2018

Dear Dr. Sescousse,

First of all, apologies for the time it has taken to deal with your paper, things tend to get a bit delayed over the holiday period. Your manuscript has now been reviewed by two external reviewers as well as by the Section Editor, Dr. Heleen Slagter, and ourselves.

Both reviewers and the editors find this to be a well-conducted and important paper, given the recent interest in spontaneous eye blink rate as a cheap, non-invasive proxy of dopamine levels in the brain. Before it can be accepted, however, please address the points raised by the reviewers in a revised version of the manuscript which we will then send back to the reviewers. In particular, please show that including global signal intensity as a co-variate of interest did not introduce the negative correlation observed. We also think it important to look into the influence of possible outliers on the results. In the revised version could you also ensure that the references are in EJN style and we will probably need higher resolution figures.

If you are able to respond fully to the points raised, we would be pleased to receive a revision of your paper within 12 weeks.

Thank you for submitting your work to EJN.

Kind regards,

Paul Bolam & John Foxe
co-Editors in Chief, EJN

Reviews:

Reviewer: 1 (Linh Dang, Vanderbilt University, USA)

Comments to the Author
The present manuscript reported no positive relationship between sEBR and dopamine synthesis capacity. As there is significant interest in finding an inexpensive, noninvasive proxy measure for dopamine function, with sEBR being the top candidate, the present findings have important implications for the field of dopamine research.

Major comments:
- Page 5 - why was global signal intensity included as a covariate of no interest? Regression of global signal intensity is known to introduce negative correlations in resting state fMRI data. I wonder if a similar phenomenon could be present here.
- Fig1A - the mask used for the region of interest analysis does not appear to have coregistered well, particularly in the caudate as shown in slice z=8
- Have the authors confirmed that there were no outliers in the data? The 2 data points with low ki values or the data point with the highest sEBR seemed like they may be outliers. Did the findings hold

without these data points?
- Fig2B - the analyses were performed with pfwe < 0.05 but the results were presented with a less rigorous threshold. The discrepancy seems to invite confusion/misinterpretation.

Minor comment:
- Page 5 - “structural MNI scans were normalized to a standard MNI template..”. Should it be “structural MRI scans”?

Reviewer: 2 (Filip Van Opstal, University of Amsterdam, Netherlands)

Comments to the Author
This study reports a PET experiment that investigates the relation between striatal dopamine synthesis capacity and spontaneous eye blink rate. Results show no support for the hypothesized positive relation. Bayesian analyses showed strong evidence in favor of the null hypothesis compared to H1 (a positive correlation) but moderate evidence for a negative relation.

The paper is well written, and the experiment competently performed and analysed.
I only have a few minor comments the authors may want to consider in a revised version of the manuscript.

Page 4: the sentence that mentions the sample rate should be rewritten.

The discussion mentions a paper that doesn’t show a positive relation between D2 receptor availability and dopamine synthesis capacity (Ito et al., 2011). It would be interesting to know if that study also hints towards a negative relation between both.

Authors’ Response 17 February 2018

Manuscript EJN-2017-11-25119, "Spontaneous eye blink rate and dopamine synthesis capacity: Preliminary evidence for an absence of positive correlation"
We thank the reviewers for their positive and constructive comments. We address these comments below and mention which changes have been made accordingly in the manuscript (colored in blue in the manuscript).

Reviewer: 1

The present manuscript reported no positive relationship between sEBR and dopamine synthesis capacity. As there is significant interest in finding an inexpensive, noninvasive proxy measure for dopamine function, with sEBR being the top candidate, the present findings have important implications for the field of dopamine research.

Major comments:

1) Page 5 - why was global signal intensity included as a covariate of no interest? Regression of global signal intensity is known to introduce negative correlations in resting state fMRI data. I wonder if a similar phenomenon could be present here.
We thank the reviewer for drawing our attention to this point. The rationale for including global signal intensity as a covariate was to ensure that any observed correlation would not originate from different infusion efficacy of [^{18}F]DOPA between participants. However, in hindsight, we realize that this is not a common procedure in neurochemical PET analyses. In fact, the potential confound related to individual differences in [^{18}F]DOPA infusion efficacy is already taken care of by using the cerebellum as a reference region and creating cerebellum-corrected Ki images. Accordingly, we removed the global intensity covariate in our voxel-wise analysis. This had very little influence on the results. The new analysis still shows a negative relationship between sEBR and Ki values in the striatum (see Figure below). In particular, this relationship still shows a significant peak in the left nucleus accumbens surviving a voxel-wise FWE corrected threshold of $p<0.05$.

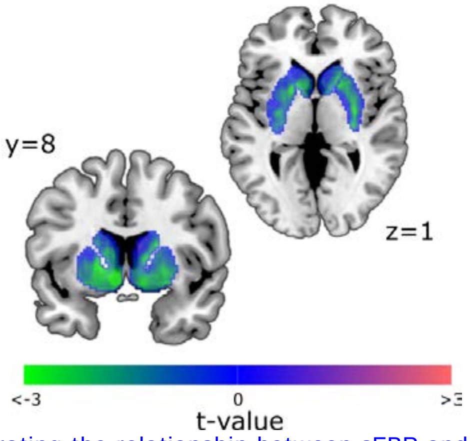

Figure: Unthresholded t-map illustrating the relationship between sEBR and Ki values in the striatum

Following the reviewer's suggestion, we have removed global intensity as a covariate in our voxel-wise analysis, and have accordingly replaced Figure 2B with the new map resulting from this analysis (now shown unthresholded, please see point #4)

2) Fig1A - the mask used for the region of interest analysis does not appear to have coregistered well, particularly in the caudate as shown in slice z=8

We see where the impression of the reviewer comes from. The mask displayed in the right panel of Figure 1A was derived from a whole-brain template of [^{18}F]DOPA binding (García-Gómez et al., 2013). In order to isolate the voxels showing maximal [^{18}F]DOPA binding (and thus corresponding to the striatum), we thresholded the template to retain the voxels exceeding the mean signal value + 3 standard deviations. Thus it is not an anatomical mask of the striatum, but a functional mask containing the voxels with maximal [^{18}F]DOPA binding in the original template. This explains why the displayed mask does not overlap perfectly with the anatomical boundaries of the striatum, even though it closely approximates the shape of the striatum. To convince the reviewer that the co-registration of the mask went well, we provide below a figure with the unthresholded [^{18}F]DOPA template overlaid on an anatomical brain including the skull, which shows that the contours of the template are well co-registered with the contours of the brain.

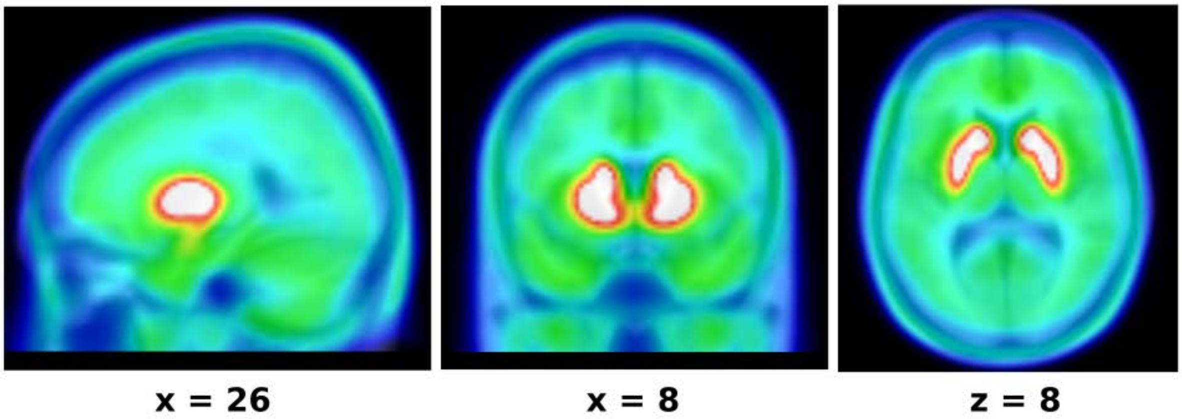

Figure: co-registration of [^{18}F]DOPA binding template onto anatomical brain template in MNI space.

We have modified the methods and legend of Figure 1 in order to better reflect that the striatal mask was defined functionally, and not anatomically:

Materials and methods (p. 5): *"For the main analyses, Ki values were extracted from an independently and functionally defined striatal region of interest, based on a whole-brain [^{18}F]DOPA PET template normalized to*

MNI space (García-Gómez et al., 2013). Specifically, we retained the template voxels in which Ki values were at least 3 standard deviations above the template mean, thus approximating the anatomical boundaries of the striatum (in which [^{18}F]DOPA binding is maximal, see Figure 1A)".

Legend of Figure 1A: *"On the right, the striatal functional mask used for the region-of-interest analyses is depicted in red".*

3) Have the authors confirmed that there were no outliers in the data? The 2 data points with low ki values or the data point with the highest sEBR seemed like they may be outliers. Did the findings hold without these data points?

We thank the reviewer for raising this point. If we remove the 3 data points flagged by the reviewer (highest Ki value and 2 lowest Ki values in the sample), the p-value of the correlation drops below significance (ρ=-0.417, p=0.096). These Ki values are 2.1 and 2.4 standard deviations away from the mean, respectively. There are thus outliers only when using the relatively stringent criterion of 2 standard deviations above/below the mean, but not when using the criterion of 2.5 standard deviations above/below the mean, which is usually deemed as a reasonable default (see e.g. Leys et al., 2013). There are no outlying sEBR values regardless of the criterion being used. In addition, we would like to emphasize that the main message of our paper does not rest on the significance of the negative correlation between sEBR and Ki values, but rather on the absence of a positive correlation (rejection of H1), as highlighted by the Bayesian statistics.

For the sake of transparency, we now mention in the legend of Figure 1 that the negative correlation between sEBR and Ki values is no longer significant if we remove the highest and two lowest Ki values, that are 2.1 and 2.4 standard deviations away from the mean, respectively (we prefer to refrain from labeling these values as outliers or not, given that we had no a priori criterion for defining outliers):

Legend of Figure 1B: *"Negative correlation between spontaneous eye blink rate and Ki values in the striatal mask (ρ=-0.504, p=0.024). The shaded area represents the 95% confidence interval. Note that the p-value of the negative correlation drops below significance (ρ=-0.417, p=0.096) when removing the 3 most extreme Ki values (highest Ki value and two lowest Ki values, respectively 2.1 and 2.4 standard deviations away from the mean)"*

4) Fig2B - the analyses were performed with pfwe < 0.05 but the results were presented with a less rigorous threshold. The discrepancy seems to invite confusion / misinterpretation.

We acknowledge the reviewer's concern. The motivation for displaying some of the results with a lenient threshold was to illustrate that the correlation between sEBR and Ki values reported to be negative in the left striatum was also negative (rather than positive) in the right striatum, albeit not significant. In order to convey this message more clearly and unambiguously, and in line with recent guidelines for improved data visualization (Allen et al., 2012; Poldrack et al., 2017), we now report an unthresholded map showing both negative and positive t-values: this map illustrates that most t-values are negative and that very few voxels display a positive correlation.

We also modified the legend of Figure 2B to avoid any confusion / misinterpretation:

Legend of Figure 2B: *"Unthresholded t-map resulting from a voxel-wise analysis investigating the relationship between sEBR and Ki values in an anatomical mask of the striatum. Note that almost all t-values (including those that do not reach statistical significance) are negative, illustrating that the relationship between sEBR and dopamine synthesis capacity is negative across a large portion of the striatum. This negative relationship shows a significant peak in the left nucleus accumbens (x, y, z = -8, 10, -6), surviving a voxel-wise FWE corrected threshold of p<0.05 within the striatal mask".*

Minor comment:

Page 5 - "structural MNI scans were normalized to a standard MNI template.". Should it be "structural MRI scans"?

We thank the reviewer for spotting this typo, we corrected it.

Reviewer: 2

This study reports a PET experiment that investigates the relation between striatal dopamine synthesis capacity and spontaneous eye blink rate. Results show no support for the hypothesized positive relation. Bayesian analyses showed strong evidence in favor of the null hypothesis compared to H1 (a positive correlation) but moderate evidence for a negative relation.

The paper is well written, and the experiment competently performed and analysed. I only have a few minor comments the authors may want to consider in a revised version of the manuscript.

Page 4: the sentence that mentions the sample rate should be rewritten.
We thank the reviewer for spotting this error, we corrected it.

The discussion mentions a paper that doesn't show a positive relation between D2 receptor availability and dopamine synthesis capacity (Ito et al., 2011). It would be interesting to know if that study also hints towards a negative relation between both.
The paper by Ito et al. (2011) indeed reports a negative correlation between striatal D2 receptor availability and dopamine synthesis capacity. However, note that compared with the study by Berry et al. (2017) which reports a positive correlation between striatal D2 receptor availability and dopamine synthesis capacity, the study by Ito et al. (2011) had a relatively low sample size (n = 14, compared with n = 40), as well a less sensitive ligand to measure dopamine synthesis capacity ([^{18}F]DOPA, compared with [^{18}F]FMT).
We have modified the Discussion to acknowledge the negative relationship reported by Ito et al. (2011) between striatal D2 receptor availability and dopamine synthesis capacity:
Discussion (p. 7): *"Also, such a relationship remains difficult to reconcile with recent findings showing a positive association between dopamine D2 receptor availability and dopamine synthesis capacity (Berry et al., 2017, but see Ito et al., 2011, showing a negative association, although in a much smaller sample size of n=14 instead of n=40), as this would predict a positive relationship between sEBR and dopamine synthesis capacity, in contrast to our results".*

References
Allen EA, Erhardt EB, Calhoun VD. Data visualization in the neurosciences: overcoming the curse of dimensionality. Neuron. 2012;74(4):603-8.
Leys C, Ley C, Klein O, Bernard P, Licata L. Detecting outliers: Do not use standard deviation around the mean, use absolute deviation around the median. Journal of Experimental Social Psychology. 2013;49(4):764-6.
Poldrack RA, Baker CI, Durnez J, Gorgolewski KJ, Matthews PM, Munafo MR, et al. Scanning the horizon: towards transparent and reproducible neuroimaging research. Nat Rev Neurosci. 2017;18(2):115-26.
